# Supplementary figures and images for: miR-214 aggravates oxidative stress in thalassemic erythroid cells by targeting ATF4
Source: PLoS One. 2024 Apr 16;19(4):e0300958. doi: 10.1371/journal.pone.0300958 (PMC11020981; doi:10.1371/journal.pone.0300958)

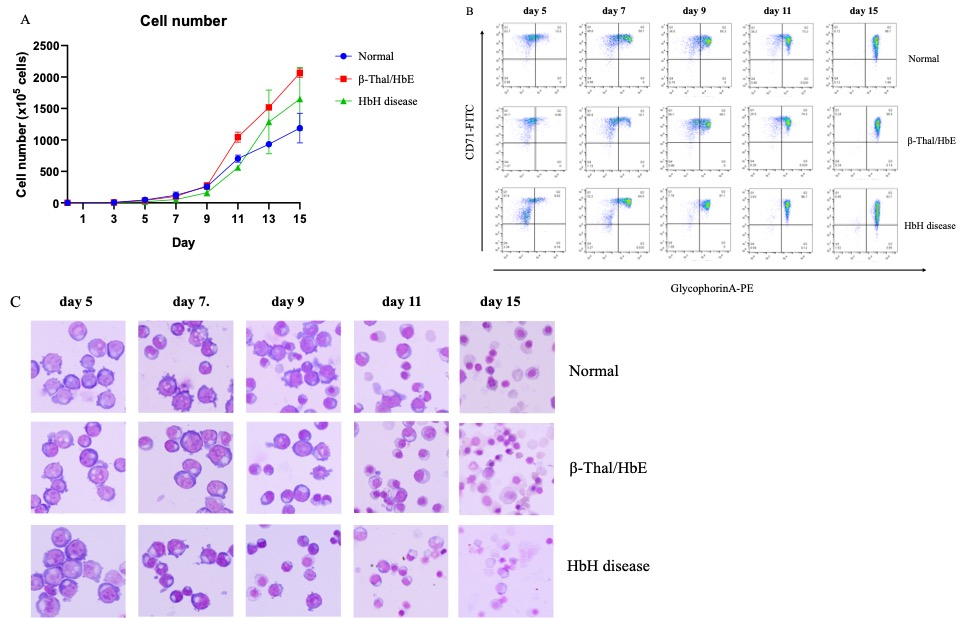

Supplement: S1 Fig — (A) Cell number of the cultured erythroid progenitor cells (B) Analysis of erythroid differentiation by flow cytometry (C) Morphology of erythroid differentiation by Wright-Giemsa staining. (TIFF) [file pone.0300958.s001.tiff]
