# Supplementary material for: miR-214 aggravates oxidative stress in thalassemic erythroid cells by targeting ATF4
Source: PLoS One. 2024 Apr 16;19(4):e0300958. doi: 10.1371/journal.pone.0300958 (PMC11020981; doi:10.1371/journal.pone.0300958)
Supplement: S1 Table — (DOCX) [file pone.0300958.s002.docx]

**S1 Table** Quantitative PCR analysis data of miR-214 expression normalized against RNU48 expression (2^-ΔCt^) after transfection with miR-214 mimic in normal samples and after transfection with anti-miR -214 inhibitor in β-thalassemia/HbE and HbH disease.

|  | Sample 1 | Sample 2 | Sample 3 |
| --- | --- | --- | --- |
| Normal Untreated control | 0.000122384 | 0.000290957 | 0.000130096 |
| Normal Negative control | 0.000108736 | 0.0002591 | 0.000311338 |
| Normal miR214 mimic | 38.17346221 | 76.90583238 | 92.18588096 |
| β-thalassemia/HbE Untreated control | 7.05119E-05 | 3.5947E-05 | 6.2789E-05 |
| β-thalassemia/HbE Negative control | 6.99448E-05 | 3.63443E-05 | 0.00010139 |
| β-thalassemia/HbE  anti-miR -214 inhibitor | 3.27895E-05 | 2.43265E-05 | 3.56072E-05 |
| HbH disease  Untreated control | 0.002834036 | 0.003730789 | 0.003264128 |
| HbH disease  Negative control | 0.002371547 | 0.004658114 | 0.00354483 |
| HbH disease  anti-miR -214 inhibitor | 0.001320979 | 0.001996203 | 0.001685909 |
