# Supplementary material for: miR-214 aggravates oxidative stress in thalassemic erythroid cells by targeting ATF4
Source: PLoS One. 2024 Apr 16;19(4):e0300958. doi: 10.1371/journal.pone.0300958 (PMC11020981; doi:10.1371/journal.pone.0300958)
Supplement: Attachment — Submitted filename: Response-to-Reviewers.docx [file pone.0300958.s004.docx]

Reviewer #1: Authors performed experiments in erythroid progenitors isolated from thalassemia Hb H and beta/Hb E patients, and normal subjects, and investigated effect of micro-RNA-214 on the erythroid by monitoring levels of expressions of mi-RNA214 and ATF4, GSH and ROS content, erythroid cell viability and apoptosis. The results are very interesting and clearly understood. I appreciate all the results and discussion; however, I would like to address my comments on the article.

Title: Yes, it's named appropriately.

Abstract and Introduction: There are some redundance that need to be modified.

Reply

Thank you for the comments and suggestion. Some redundance was modified.

Methods: I suggest the authors introduce details of the excitation and emission wavelengths used in flow cytometry of GSH and ROS, important!!

Reply We had added the details of the excitation and emission wavelengths. This had been indicated in Materials and methods part, page 9, line 180-182 and line 188-189, 201 and page 10, line 203-204.

References: Use "Sci World J" instead of "Scientific World Journal".

Reply Thank you for the comments and suggestion. The reference had been corrected.

Figure illustration: x-Axis, y-axis and graph lines in figures 6-9 are not clear, please improve their quality.

Reply Thank you for the comments and suggestion. We had improved the picture quality.
